# Factors Prognostic of Survival in Liver Transplant Recipients with Hepatitis B Virus Related Acute-on-Chronic Liver Failure

**DOI:** 10.1155/2022/6390809

**Published:** 2022-05-10

**Authors:** Zhengjun Zhou, Junfang Yi, Qiang Li, Wei Hu, Guangshun Chen, Zhongzhou Si, Jiequn Li

**Affiliations:** ^1^Department of Liver Transplant, Second Xiangya Hospital, Central South University, Changsha, Hunan 410011, China; ^2^Department of Hepatobiliary Surgery, Xijing Hospital, Fourth Military Medical University, Xi'an, Shaanxi 710032, China; ^3^Transplant Medical Research Center, Second Xiangya Hospital, Central South University, Changsha, Hunan 410011, China

## Abstract

**Objectives:**

Factors prognostic of survival in liver transplant (LT) recipients with hepatitis B virus related acute-on-chronic liver failure (HBV-ACLF) remain unclear. This study evaluated risk factors for survival in LT recipients with HBV-ACLF and determined the scoring system optimal for assessing patient prognosis.

**Methods:**

This retrospective study included 323 HBV-ACLF related patients undergoing LT, including 112, 146, and 65 patients with HBV-ACLF grades 1, 2, and 3, respectively. Overall survival (OS) was estimated by the Kaplan–Meier method, and factors associated with survival were analysed by multivariate Cox proportional hazards models. Pretransplant prognostic scoring systems were compared by receiver operating characteristic (ROC) curve analysis.

**Results:**

The one-year survival rate was significantly lower in HBV-ACLF grade 3 (80.0%) than in grades 1 (93.8%) and 2 (91.8%) recipients (*p*=0.0063). Cox multivariate analysis showed that age >53 years (hazard ratio (HR) 3.731; 95% confidence interval (CI) 1.640–8.407), WBC count >8.6 × 10^9^/L (HR 4.544; 95% CI 1.140–18.107), HBV-ACLF 3 (HR 2.729; 95% CI 1.050–7.096), and cold ischaemia time >8.5 hours (HR 2.867; 95% CI, 1.38–5.921) were independently prognostic of 1-year survival. Comparisons of pretransplant scoring systems showed that chronic liver failure-consortium ACLF score (CLIF-C ACLFs) was superior to COSSH-ACLF, MELD-Na, and MELD scores in predicting 1-year OS in these patients.

**Conclusions:**

Age >53 years, WBC counts >8.6 × 10^9^/L, HBV-ACLF grade 3, and cold ischaemia time >8.5 hours are independently prognostic of OS in LT recipients with HBV-ACLF. CLIF-C ACLFs is superior to other scoring methods in predicting 1-year OS in these patients.

## 1. Introduction

Hepatitis B virus related acute-on-chronic liver failure (HBV-ACLF) is a complex syndrome characterised by acute deterioration of liver function and high mortality rates, resulting in hepatic and extrahepatic organ failure due to chronic hepatitis B, regardless of the presence of cirrhosis [1, 2]. Although some ACLF patients have autoimmune hepatitis, most patients with ACLF in the Asia-Pacific region have HBV-associated ACLF patients [3–6]. Short-term mortality rates of HBV-ACLF patients receiving comprehensive conservative management have been reported to be as high as 50% to 90% [2, 7]. At present, liver transplantation (LT) is the most effective treatment for patients with HBV-ACLF [8–12]. Although factors prognostic of LT in patients with ACLF have been widely reported, few studies to date have focused on HBV-related ACLF and assessed the post-LT prognosis of patients with HBV-ACLF; moreover, those studies included relatively small numbers of patients and did not completely evaluate indicators [12, 13]. No studies to date have reported reliable and uniform prognostic factors that can predict survival outcomes in LT recipients who developed HBV-ACLF.

Several prognostic scoring systems have been formulated to evaluate LT-free survival of patients with HBV-ACLF, including the chronic liver failure-consortium ACLF (CLIF-C ACLF) score [14], the Chinese group on the study of severe hepatitis B-ACLF (COSSH-ACLF) score [2], the COSSH-ACLF II score [1], the model for end-stage liver disease (MELD) score [15], and the MELD-sodium (MELD-Na) score [16]. Although patients in China are usually evaluated by determining their COSSH-ACLF II scores [1], the optimum scoring system for evaluating outcomes of LT recipients who developed HBV-ACLF has not been determined. The present study therefore evaluated the risk factors for survival in HBV-ACLF patients following LT and determined the scoring system optimal for assessing patient prognosis.

## 2. Methods

### 2.1. Study Population

**Figure 1:**
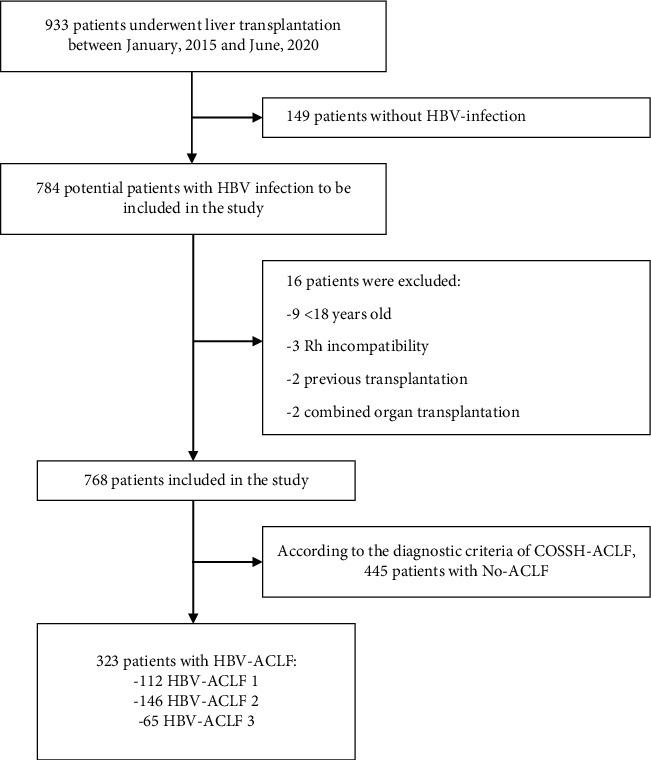
Flow chart of the patients included in this study.

All patients (*N* = 933) who underwent LT in the Second Xiangya Hospital, Central South University between January 2015 and June 2020 were retrospectively evaluated (Figure 1). Patients not infected with HBV (*N* = 149) were excluded, as were patients aged <18 years, those with Rh incompatibility, and those who had undergone previous LT and combined organ transplantation (*N* = 16). HBV-infected patients who did not develop ACLF were also excluded from the study (*N* = 445). The 323 LT recipients with HBV-ACLF included in this study were divided into three grades based on the number of failed organs [2], with 112, 146, and 65 patients classified as having HBV-ACLF grades 1, 2, and 3, respectively. All patients were followed up for more than 1 year after LT. The study protocol conformed to the ethical guidelines of the Declaration of Helsinki and was approved by the institutional review board of the Second Xiangya Hospital, Central South University (No. 2019-050). A written informed consent was obtained from all the participants.

### 2.2. Operative Procedures and Immunosuppression Therapy

Starting from 1 January 2015, all organ donations were performed according to the Chinese protocol [17]. Hepatic allografts were obtained using a rapid technique for the procurement of donor livers, including flushing of the aortic and portal veins and cold storage, as described previously [18]. All hepatic allografts were perfused with and preserved in the University of Wisconsin (UW) solution. All operative procedures were performed using standard techniques or piggyback methods. Patients undergoing LT received ATG-Fresenius or basiliximab and methylprednisolone as part of the induction protocol. Standard posttransplant immunosuppressive treatment included tacrolimus and mycophenolate mofetil.

### 2.3. Statistical Analysis

Continuous variables were reported as medians (ranges) and categorical variables as numbers (percentages (%)). Groups were compared using Kruskal–Wallis tests, unpaired Student's *t*-tests, and Pearson's chi-squared or Fisher's exact tests, as warranted. Overall survival (OS) was estimated by the Kaplan–Meier method. Receiver operating characteristic (ROC) curves were calculated for the MELD, MELD-Na, CLIF-C ACLF, COSSH-ACLF, and COSSH-ACLF preoperative prognostic scoring systems, and the areas under the ROC curves were compared using *Z* tests (Delong's method [19]). Risk factors significantly affecting patient survival were determined and included in a Cox proportional hazards regression model analysis. All statistical analyses were performed using SPSS 11.0 (IBM Corp., USA) or MedCalc 15.0 (MedCalc Software Ltd, Belgium) statistical software, with *p* values < 0.05 considered statistically significant.

## 3. Results

### 3.1. General Characteristics of LT Recipients with HBV-ACLF

**Figure 2:**
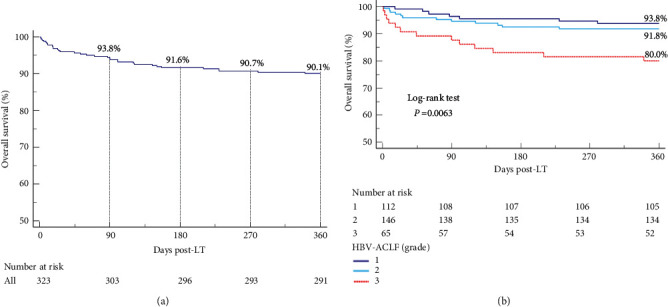
One-year overall survival of LT recipients who developed HBV-ACLF. One-year overall survival rates of (a) all recipients with HBV-ACLF and of (b) all recipients stratified by ACLF grade at LT.

A total of 323 HBV-ACLF related patients undergoing LT were eligible for inclusion in the study. Their 90-, 180-, 270-, and 360-day OS rates following LT were 93.8%, 91.6%, 90.7%, and 90.1%, respectively (Figure 2(a)). The one-year survival rate was significantly lower in recipients with HBV-ACLF grade 3 (80.0%) than in those with HBV-ACLF grades 1 (93/8%) and 2 (91.8%, *p*=0.0063; Figure 2(b)).

Table 1 describes the demographic and clinical characteristics of the study population. Recipients with HBV-ACLF 1, HBV-ACLF 2, and HBV-ACLF 3 were similar in age, gender distribution, the incidence of ascites, and serum concentrations of total bilirubin, urea, and sodium. However, significant differences were observed in body mass index (BMI), total creatinine concentration, international normalised ratio (INR), white blood cell (WBC), neutrophil and platelet counts, and haemoglobin concentrations, with all of these parameters being significantly worse in the HBV-ACLF grade 3 than in the other two groups. As expected, preoperative scores were significantly higher in the HBV-ACLF 3 than in the HBV-ACLF 1 and HBV-ACLF 2 groups (*p* < 0.001). The baseline characteristics and laboratory data immediately before liver procurement did not differ among donors to patients in the three HBV-ACLF groups.

### 3.2. Univariate Analysis of Factors Associated with Prognosis in LT Recipients with HBV-ACLF

The clinical characteristics of LT recipients with HBV-ACLF who did and did not survive for 1 year after LT were compared (Table 2). Although gender distribution, BMI, incidence of ascites, total bilirubin, creatinine, sodium, and haemoglobin concentrations, INR, and platelet counts did not differ significantly in these two groups, significant differences were observed in patient age, serum urea concentration, WBC and neutrophil counts, and HBV-ACLF grade. Pretransplant scores, except for MELD score, were significantly higher in patients who died than in those who survived. Donors to patients in these two groups showed no significant differences in age, gender distribution, BMI, proportions with fatty liver, donation after cardiac death, cardiopulmonary resuscitation, and laboratory data. The rate of hepatitis B surface antigen (HBsAg) positivity was significantly lower and cold ischaemia time was significantly shorter in donors of patients who survived than in those who died.

### 3.3. Factors Prognostic of Survival in LT Recipients with HBV-ACLF

**Figure 3:**
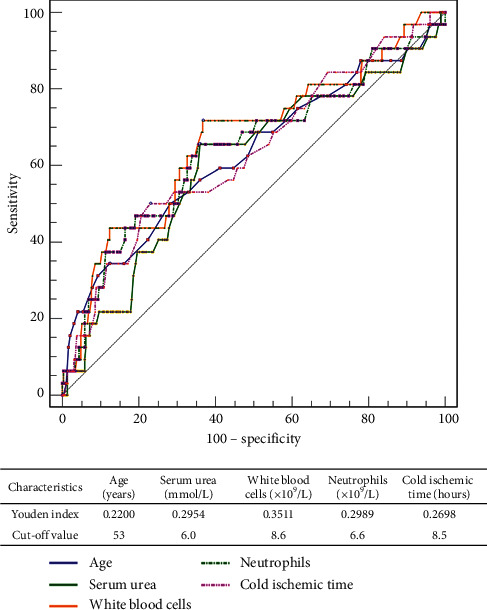
ROC curve and Youden index analyses of risk factors for survival in patients with HBV-ACLF following LT. Optimal cut-off values were determined by Youden index analysis.

Univariate analysis showed that age, serum urea concentration, WBC count, and neutrophil count of recipients, as well as cold ischaemia time of donors, were risk factors significantly affecting the prognosis of LT recipients with HBV-ACLF (Table 2). Patients were followed up for 1 year after LT, with death being the final outcome. The ROC curve analysis showed the cut-off values for these indicators (Figure 3), with the optimal cut-off values determined by calculating the maximum area under the ROC (AUROC) curve and the corresponding Youden index. These cut-off values included age 53 years, serum urea concentration 6 mmol/L, WBC count 8.6 × 10^9^/L, neutrophil count 6.6 × 10^9^/L, and cold ischaemia time 8.5 hours.

These cut-off values, along with HBV-ACLF grade and HBsAg, were included in Cox univariate and multivariate analyses of factors significantly associated with patient survival 1 year after LT (Table 3). Cox multivariate analysis showed that age >53 years (hazard ratio (HR) 3.731; 95% confidence interval (CI) 1.640–8.407), WBC count >8.6 × 10^9^/L (HR 4.544; 95% CI 1.140–18.107), HBV-ACLF 3 (HR 2.729; 95% CI 1.050–7.096), and cold ischaemia time >8.5 hours (HR 2.867; 95% CI 1.38–5.921) were independently prognostic of 1-year survival in these LT recipients.

### 3.4. Survival Analysis of HBV-ACLF Recipients after LT

**Figure 4:**
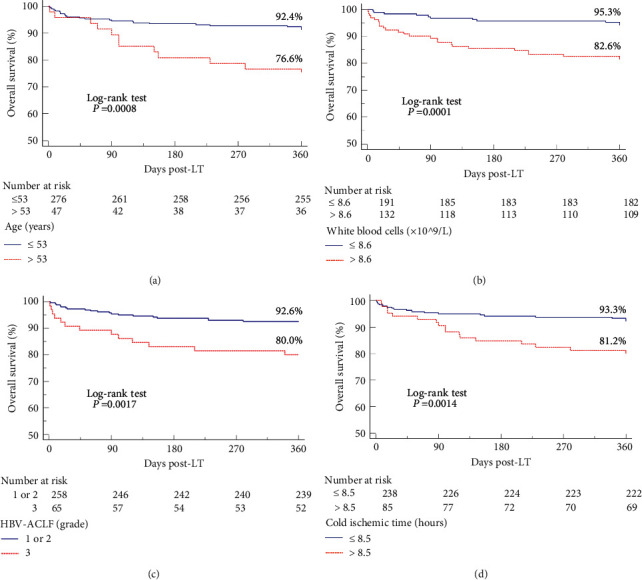
Kaplan–Meier analyses of 1-year survival rates in recipients with HBV-ACLF (a) aged >53 years (vs. ≤53 years), (b) WBC counts >8.6 × 109/L (vs. ≤8.6 × 109/L), (c) HBV-ACLF grade 3 (vs. HBV-ACLF grades 1 and 2), and (d) cold ischaemia time >8.5 hours (vs. ≤8.5 hours) (*p* < 0.01).

Kaplan–Meier analysis of survival in HBV-ACLF recipients after LT (Figure 4) showed that 1-year survival rates were significantly lower in patients aged >53 than ≤53 years (72.6% vs. 92.4%, *p*=0.0008) (Figure 4(a)), in patients with WBC counts >8.6 × 109/L than ≤8.6 (82.6% vs. 95.3%, *p*=0.0001) (Figure 4(b)), in patients with HBV-ACLF grade 3 than in those with HBV-ACLF grades 1 and 2 (80.0% vs. 92.6%, *p*=0.0017) (Figure 4(c)), and in patients with cold ischaemia times >8.5 hours than ≤8.5 hours (81.2% vs. 93.3%, *p*=0.0014) (Figure 4(d)).

### 3.5. Comparison of Prognostic Scores in LT Recipients with HBV-ACLF

**Figure 5:**
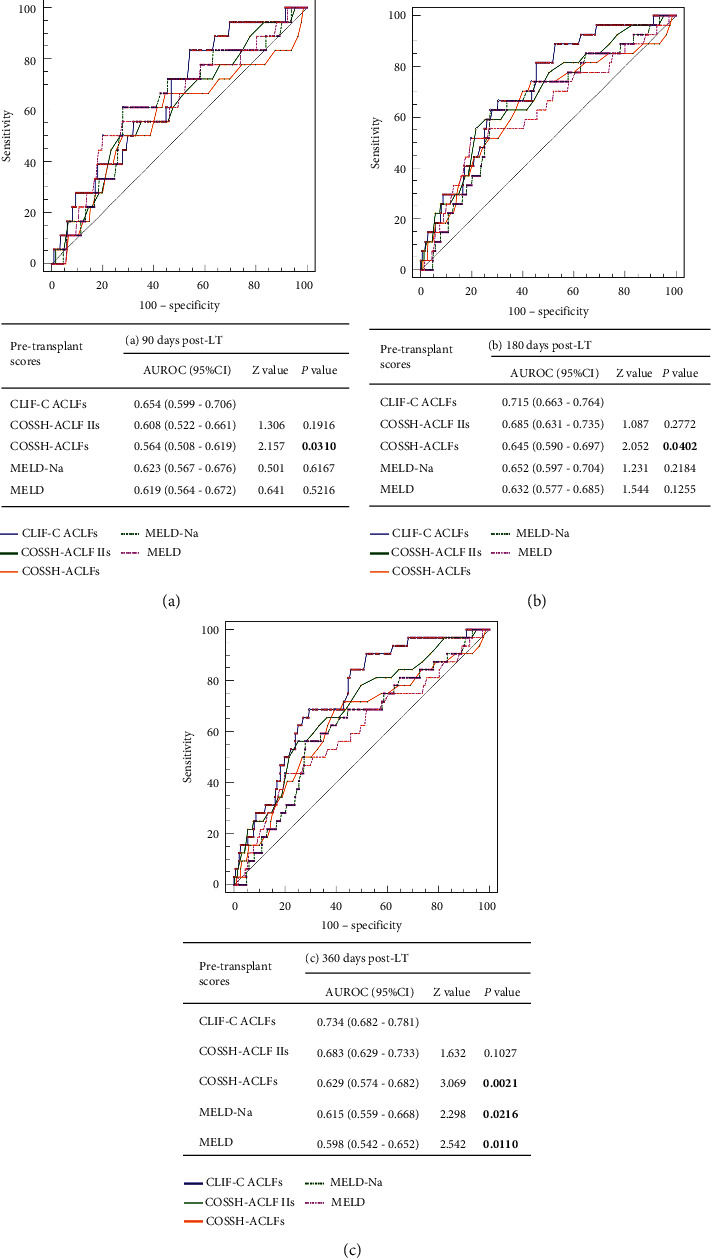
ROC curves and AUROC comparisons of the five pretransplant scoring systems predicting the survival probability of patients with HBV-ACLF (a) 90, (b) 180, and (c) 360 days after LT.

All pretransplant scores were significantly higher in patients who survived than in those who died (Table 2). The five pretransplant scores were compared using ROC curves and *Z* tests to determine which optimally predicted survival after LT. The CLIF-C ACLF scoring system was significantly more accurate in predicting patient prognosis than the COSSH-ACLF IIs, COSSH-ACLF, MELD-Na, and MELD scores. The AUROCs of the CLIF-C ACLF score for predicting mortality at 90, 180, and 360 days in patients with HBV-ACLF after LT were 0.654, 0.715, and 0.734, respectively (Figure 5).

## 4. Discussion

This study analysed the survival of HBV-ACLF patients following LT at a single centre in China from January 2015 to June 2020. Of the 933 patients who underwent LT, 323 (34.6%) had HBV-ACLF, with these patients having a 1-year survival rate of 90.1%. LT was shown to be safe and effective, with good outcomes in patients with HBV-ACLF. The present study found that several risk factors, involving both recipients and donors, were associated with recipient prognosis. Recipient factors included age, serum urea concentration, WBC and neutrophil counts, pretransplant scores, and HBV-ACLF grade, whereas donor factors included HBsAg positivity and cold ischaemia time. Cox multivariate and Kaplan–Meier survival analyses showed that recipient age, WBC counts, and HBV-ACLF grade, along with donor cold ischaemia time, were significantly prognostic of survival in LT recipients with HBV-ACLF. In addition, a comparison of pretransplant scores found that the CLIF-C ACLF scoring system was more accurate than the other scoring systems in evaluating survival outcomes in these patients.

Several previous studies have evaluated factors associated with prognosis of HBV-ACLF patients following LT. For example, a study of 290 patients with ACLF after LT found that recipient WBC count, the ratio of alanine aminotransferase (ALT) to aspartate aminotransferase (AST), and the number of organs that failed were prognostic of survival [13]. Another study, of 78 patients, reported that patients with initial COSSH-ACLF grade 3 and with grades 2 and 3 on days 3–7 after diagnosis had poorer prognosis [12]. In these studies, HBV-ACLF was diagnosed using the Asian Pacific association for the study of the liver (APASL) criteria [6], which did not include donor characteristics. In addition, the risk of death after LT was found to be higher in patients with ACLF 3 who required mechanical ventilation at LT and who received marginal organs [20]. That study, however, excluded patients diagnosed with HBV-ACLF.

In the present study, characteristics of both recipients and donors were evaluated to determine factors prognostic of survival in HBV-ACLF patients who underwent LT. Consistent with previous findings, the present study found that recipient age was an important prognostic factor in liver transplant patients [21, 22], in that the one-year survival rate was significantly lower in HBV-ACLF patients aged >53 than ≤53 years. In addition, pretransplant infection has been reported to affect the posttransplant fatality rate in ACLF patients who underwent LT [23]. The present study found that HBV-ACLF patients with WBC counts over 8.6 × 10^9^/L had poorer outcomes after LT than those with WBC counts ≤8.6 × 10^9^/L.

The severity of disease in ACLF patients undergoing LT is a key factor affecting prognosis [11, 20]. The present study found that the 1-year OS rate was significantly lower in patients with ACLF grade 3 than in those with ACLF grades 1 and 2. However, the 1-year OS rate in patients with ACLF grade 3 was 80% after LT, significantly higher than in patients with ACLF grade 3 who did not undergo LT [1, 2, 12, 24, 25]. These findings suggested that patients with HBV-ACLF be assessed for LT, with indications determined on an emergency basis, particularly for patients with a severe early course.

Although univariate analysis showed that receiving grafts from HBsAg-positive donors was a risk factor for HBV-ACLF patients, this factor was not statistically significant on multivariate analysis and was not independently prognostic of survival. This finding is consistent with results showing that receiving grafts from HBV positive donors did not increase complication or mortality rates after LT [26]. Additionally, mortality rates were found to be significantly higher in HBV-ACLF patients with cold ischaemia times >8.5 hours than ≤8.5 hours throughout the entire observation period, suggesting that longer cold ischaemia time has a major impact on the prognosis of HBV-ACLF patients after LT.

Similar to our findings, previous studies have reported that the CLIF-C ACLF scoring system was superior to the classical MELD and MELD-Na scores in evaluating the prognosis of patients with ACLF [14, 25]. In contrast, other studies have suggested that the COSSH-ACLF scoring system was better than the CLIF-C ACLF system in evaluating the outcomes of patients with HBV-ACLF [1, 2]. All of these studies, however, evaluated outcomes in ACLF patients who did not undergo LT. Based on these findings, the present study used the COSSH-ACLF scoring system to select and classify HBV-CALF recipients. Compared with the APASL and EASL-ACLF scoring systems, the COSSH-ACLF scoring system identified nearly 20% more patients with HBV-ACLF, thus increasing their opportunity to get timely intensive management and earlier LT [1, 2]. The present study showed that the CLIF-C ACLF scoring system was superior to the four other pretransplant scoring systems in evaluating patient prognosis after LT, including survival rates at 90, 180, and 360 days.

This study had several limitations, including its retrospective design and its inclusion of a relatively small number of patients who underwent LT at a single transplant centre. Prospective, multicentre studies with a larger sample size are needed to optimise the ability to predict the prognosis of LT in patients with HBV-ACLF.

## 5. Conclusions

Overall, this study evaluated risk factors affecting the prognosis of HBV-ACLF patients undergoing LT, finding that recipient age, WBC count, HBV-ACLF grade, and cold ischaemia time were significantly prognostic in this patient cohort. Pretransplant CLIF-C ACLF score was predictive of survival outcomes after LT in patients with HBV-ACLF.

## Figures and Tables

**Table 1:** Demographic and clinical characters of liver transplant donors and recipients, categorised by HBV-ACLF grade.

Characteristics	HBV-ACLF 1	HBV-ACLF 2	HBV-ACLF 3	*p* value
	(*n* = 112)	(*n* = 146)	(*n* = 65)	
Recipient				
Age (years)	47.0 (22.0–70.0)	45.5 (22.0–62.0)	44.0 (23.0–62.0)	0.088
Male, *n* (%)	106 (94.6)	134 (91.8)	61 (93.8)	0.646
Body mass index (kg/m^2^)	22.7 (15.0–32.4)	23.8 (17.3–35.9)^*∗*^	24.2 (16.3–34.0)^*∗*^	0.011
Ascites, *n* (%)	89 (79.5)	109 (74.7)	44 (67.7)	0.218
Laboratory data				
Total bilirubin (*μ*mol/L)	431.2 (87.7–842.6)	447.9 (195.3–850.9)	405.2 (210.4–775.5)	0.167
Serum creatinine (*μ*mol/L)	65.1 (36.6–156.0)	71.8 (23.0–725.1)	77.7 (36.0–633.1)^*∗*^	0.012
Serum urea (mmol/L)	5.3 (1.5–19.3)	5.5 (0.9–56.5)	5.6 (1.0–49.4)	0.977
International normalised ratio	2.0 (1.0–3.4)	3.1 (1.5–8.2)^*∗*^	3.5 (1.5–14.7)^*∗*^	0.000
Serum sodium (mmol/L)	136.9 (114.2–156.9)	137.0 (120.2–146.2)	1367.2 (121.3–153.7)	0.722
White blood cells (×10^9^/L)	5.9 (1.4–16.2)	7.7 (2.2–27.5)^*∗*^	10.4 (2.6–42.6)^*∗*^^,^^*∗∗*^	0.000
Neutrophils (×10^9^/L)	4.3 (0.9–13.4)	5.6 (1.3–21.7)^*∗*^	8.1 (0.3–39.3)^*∗*^^,^^*∗∗*^	0.000
Haemoglobin (g/L)	98.5 (59.0–148.0)	101.5 (62.0–164.0)	113.0 (52.0–157.0)^*∗*^^,^^*∗∗*^	0.000
Platelets (×10^9^/L)	65.0 (13.0–292.0)	57.5 (9.0–256.0)	78.0 (12.0–300.0)^*∗∗*^	0.036
Pretransplant scores				
MELD	23.5 (13.9–34.7)	30.9 (18.1–48.0)^*∗*^	33.3 (20.3–48.2)^*∗*^	0.000
MELD-Na	24.0 (13.9–43.2)	31.5 (18.4–66.4)^*∗*^	34.2 (20.3–62.5)^*∗*^	0.000
CLIF-C ACLFs	38.2 (24.4–51.0)	45.6 (28.5–60.8)^*∗*^	53.1 (44.9–65.6)^*∗*^^,^^*∗∗*^	0.000
COSSH-ACLFs	6.2 (4.9–8.2)	7.6 (5.6–12.0)^*∗*^	8.6 (6.9–16.3)^*∗*^^,^^*∗∗*^	0.000
COSSH-ACLF IIs	7.0 (5.2–9.0)	8.2 (6.3–10.5)^*∗*^	9.0 (7.0–11.6)^*∗*^^,^^*∗∗*^	0.000

Donor				
Age (years)	46.0 (6.0–69.0)	44.5 (9.0–70.0)	47.0 (12.0–66.0)	0.260
Male, *n* (%)	89 (79.5)	127 (87.0)	55 (84.6)	0.261
Body mass index (kg/m^2^)	22.9 (14.8–59.0)	23.1 (12.9–33.4)	22.5 (13.8–32.3)	0.979
Fatty liver, *n* (%)	26 (23.2)	31 (21.2)	21 (32.3)	0.213
HBsAg positive, *n* (%)	10 (8.9)	20 (13.7)	5 (7.8)	0.323
Donation after cardiac death, *n* (%)	3 (2.7)	4 (2.7)	2 (3.1)	0.987
Cardiopulmonary resuscitation, *n* (%)	17 (15.2)	24 (16.4)	12 (18.5)	0.851
Laboratory data				
Total bilirubin (*μ*mol/L)	11.9 (2.2–81.9)	15.1 (3.7–59.4)	14.2 (2.4–71.4)	0.053
Serum creatinine (*μ*mol/L)	89.9 (17.6–555.8)	85.8 (16.0–648.0)	105.5 (28.0–396.6)	0.116
International normalised ratio	1.1 (0.8–7.3)	1.1 (0.8–3.0)	1.0 (0.8–2.5)	0.867
Serum sodium (mmol/L)	145.0 (128.0–182.1)	145.8 (123.1–195.0)	144.5 (128.0–178.0)	0.499
Cold ischaemia time (hours)	7.4 (3.2–13.4)	7.8 (0.9–12.2)	8.0 (3.2–12.1)	0.245

Abbreviations: MELD, model for end-stage liver disease score; MELD-Na, MELD-sodium score; CLIF-C ACLF, chronic liver failure-consortium ACLF score; COSSH-ACLF, Chinese group on the study of severe hepatitis B-ACLF score; COSSH-ACLF II, Chinese group on the study of severe hepatitis B-ACLF II score. ^*∗*^*p* < 0.05 vs. HBV-ACLF 1; ^*∗∗*^ *p* < 0.05 vs. HBV-ACLF 2.

**Table 2:** Univariate analysis of factors associated with survival after LT in recipients with HBV-ACLF.

Characteristics	Survived (*n* = 291)	Died (*n* = 32)	*p* value
Recipient			
Age (years)	45.0 (22.0–70.0)	50.5 (24.0–62.0)	0.019
Male, *n* (%)	271 (93.1)	30 (93.8)	0.625
Body mass index (kg/m^2^)	23.4 (15.0–35.9)	24.6 (17.3–28.7)	0.301
Ascites, *n* (%)	216 (74.2)	26 (81.3)	0.262
Laboratory data			
Total bilirubin (*μ*mol/L)	434.6 (87.7–850.9)	418.7 (233.0–747.7)	0.717
Serum creatinine (*μ*mol/L)	68.5 (23.0–725.1)	84.1 (34.5–529.3)	0.054
Serum urea (mmol/L)	5.4 (0.9–56.5)	6.7 (1.2–36.9)	0.048
International normalised ratio	2.6 (1.0–14.7)	2.9 (1.5–6.5)	0.190
Serum sodium (mmol/L)	137.0 (114.2–156.9)	135.6 (122.9–144.3)	0.132
White blood cells (×10^9^/L)	7.2 (1.4–37.6)	10.0 (2.9–42.6)	0.002
Neutrophils (×10^9^/L)	5.3 (0.9–33.5)	7.8 (0.3–39.3)	0.008
Haemoglobin (g/L)	103.0 (52.0–164.0)	106.5 (69.0–143.0)	0.585
Platelets (×10^9^/L)	65.0 (9.0–292.0)	75.0 (25.0–300.0)	0.080
Grade of HBV-ACLF, *n* (%)			0.008
HBV-ACLF 1	105 (93.8)	7 (6.2)	
HBV-ACLF 2	134 (91.8)	12 (8.2)	
HBV-ACLF 3	52 (80.0)	13 (20.0)	
Pretransplant scores			
MELD	27.9 (13.9–48.2)	31.3 (18.1–46.9)	0.068
MELD-Na	29.8 (13.9–66.4)	34.5 (20.1–48.6)	0.033
CLIF-C ACLFs	43.6 (24.4–63.8)	49.4 (33.7–65.6)	0.000
COSSH-ACLFs	7.1 (4.9–16.3)	8.0 (5.4–12.2)	0.017
COSSH-ACLF IIs	7.8 (5.2–11.6)	8.8 (6.5–11.4)	0.001

Donor			
Age (years)	46.0 (6.0–70.0)	37.0 (15.0–60.0)	0.062
Male, *n* (%)	243 (83.5)	28 (87.5)	0.387
Body mass index (kg/m^2^)	23.0 (12.9–59.0)	22.1 (15.2–31.3)	0.187
Fatty liver, *n* (%)	73 (25.1)	5 (15.6)	0.235
HBsAg positive, *n* (%)	27 (9.3)	8 (25.0)	0.013
Donation after cardiac death, *n* (%)	7 (2.4)	2 (6.3)	0.210
Cardiopulmonary resuscitation, *n* (%)	47 (16.2)	6 (18.8)	0.706
Laboratory data			
Total bilirubin (*μ*mol/L)	14.0 (2.2–81.9)	12.4 (4.4–25.0)	0.274
Serum creatinine (*μ*mol/L)	89.5 (16.0–648.0)	70.1 (31.0–376.0)	0.402
International normalised ratio	1.1 (0.8–3.0)	1.1 (0.9–7.3)	0.280
Serum sodium (mmol/L)	145.7 (123.1–195.0)	143.1 (128.1–173.0)	0.476
Cold ischaemia time (hours)	7.5 (0.9–13.4)	8.5 (4.8–12.1)	0.016

**Table 3:** Univariate and multivariate Cox proportional hazards models of factors associated with post-LT survival in HBV-ACLF patients.

Characteristics	Reference	Hazard ratio^†^ (95.0% CI)	*p* value	Hazard ratio^‡^ (95.0% CI)	*p* value
Recipient					
Age >53 years	≤53	3.244 (1.564–6.730)	0.002	3.713 (1.640–8.407)	0.002
Serum urea >6.0 mmol/L	≤6.0	3.146 (1.517–6.525)	0.002	1.978 (0.900–4.439)	0.090
White blood cells >8.6 × 10^9^/L	≤8.6	3.974 (1.839–8.589)	0.000	4.544 (1.140–18.107)	0.032
Neutrophils >6.6 × 10^9^/L	≤6.6	3.069 (1.480–6.366)	0.003	0.722 (0.196–2.667)	0.625
HBV-ACLF 2	1	1.339 (0.527–3.401)	0.539	1.433 (0.531–3.871)	0.478
HBV-ACLF 3	1	3.496 (1.395–8.764)	0.008	2.729 (1.050–7.096)	0.039

Donor					
HBsAg positive	Negative	2.850 (1.280–6.344)	0.010	2.328 (0.994–5.455)	0.052
Cold ischaemia time >8.5 hours	≤8.5	2.928 (1.464–5.855)	0.002	2.867 (1.389–5.921)	0.004

Notes: ^†^Univariable analysis, ^‡^Multivariable analysis.

## Data Availability

The data used to support the findings of this study are available from the corresponding author upon reasonable request.
